# Exposure to anti-malarial drugs and monitoring of adverse drug reactions using toll-free mobile phone calls in private retail sector in Sagamu, Nigeria: implications for pharmacovigilance

**DOI:** 10.1186/1475-2875-10-230

**Published:** 2011-08-09

**Authors:** Ahmed A Adedeji, Bilqees Sanusi, Azeez Tella, Motunrayo Akinsanya, Olubusola Ojo, Mufliat O Akinwunmi, Olubukola A Tikare, Isiaka A Ogunwande, Omobola A Ogundahunsi, Olajide O Ayilara, Taofeeqah T Ademola, Fatai A Fehintola, Olumide AT Ogundahunsi

**Affiliations:** 1 Communicable Disease Research Unit, Olabisi Onabanjo University Teaching Hospilal Sagamu, PMB 2002, Nigeria; 2 Department of Pharmacology, Olabisi Onabanjo University, PMB 2022, Sagamu, Nigeria; 3 Faculty of Pharmacy, Olabisi Onabanjo University, PMB 2022, Sagamu, Nigeria; 4 Department of Pharmacology and Toxicology, Kampala International University- Western Campus, PO Box 71, Ishaka Bushenyi District, Uganda; 5 The School of Pharmacy, University of London, 29-39 Brunswick Square, London WC1N 1AX, UK; 6 Department of Chemistry, Faculty of Science, Lagos State University, Ojo, Lagos, Nigeria; 7 Department of Pharmacology and Therapeutics, College of Medicine, University of Ibadan, Ibadan, Nigeria; 8 UNICEF/UNDP/World Bank/WHO Special Programme for Research and Training on Tropical Diseases, Geneva, Switzerland

## Abstract

**Background:**

Adverse drug reactions (ADRs) contribute to ill-health or life-threatening outcomes of therapy during management of infectious diseases. The exposure to anti-malarial and use of mobile phone technology to report ADRs following drug exposures were investigated in Sagamu - a peri-urban community in Southwest Nigeria.

**Methods:**

Purchase of medicines was actively monitored for 28 days in three Community Pharmacies (CP) and four Patent and Proprietary Medicine Stores (PPMS) in the community. Information on experience of ADRs was obtained by telephone from 100 volunteers who purchased anti-malarials during the 28-day period.

**Results and Discussion:**

A total of 12,093 purchases were recorded during the period. Antibiotics, analgesics, vitamins and anti-malarials were the most frequently purchased medicines. A total of 1,500 complete courses of anti-malarials were purchased (12.4% of total purchases); of this number, purchases of sulphadoxine-pyrimethamine (SP) and chloroquine (CQ) were highest (39.3 and 25.2% respectiuvely). Other anti-malarials purchased were artesunate monotherapy (AS) - 16.1%, artemether-lumefantrine (AL) 10.0%, amodiaquine (AQ) - 6.6%, quinine (QNN) - 1.9%, halofantrine (HF) - 0.2% and proguanil (PR) - 0.2%. CQ was the cheapest (USD 0.3) and halofantrine the most expensive (USD 7.7). AL was 15.6 times ($4.68) more expensive than CQ. The response to mobile phone monitoring of ADRs was 57% in the first 24 hours (day 1) after purchase and decreased to 33% by day 4. Participants in this monitoring exercise were mostly with low level of education (54%).

**Conclusion:**

The findings from this study indicate that ineffective anti-malaria medicines including monotherapies remain widely available and are frequently purchased in the study area. Cost may be a factor in the continued use of ineffective monotherapies. Availability of a toll-free telephone line may facilitate pharmacovigilance and follow up of response to medicines in a resource-poor setting.

## Background

Infection with malaria parasites remains one of the leading causes of hospital visits in Nigeria. Half of the estimated population of 150 million experience at least one malarial attack each year [1]. Early diagnosis and treatment of malaria is well recognized as an effective means of reducing morbidity and mortality of the infection [2]. Chloroquine (CQ) and sulphadoxine-pyrimethamine were cheap and effective treatment for malaria introduced in the middle of the 20^th ^century. Unfortunately emergence of *Plasmodium falciparum *resistance to these drugs in South East Asia and eventual spread to all malaria endemic areas compromised malaria treatment of falciparum malaria in several malaria endemic countries, including Nigeria [3] Artemisinin-based combination therapy (ACT) was subsequently recommended by the WHO and introduced as part of a multi-component strategy to mitigate the malaria burden [2-4]. As ACT become more widely available and accessible, there is a need to assess and document experience and occurrence of adverse drug reactions (ADRs) of these drugs when used in larger populations or communities [5]. In the industrialized economies of the northern hemisphere, relatively robust health systems with capability for systematic acquisition of post marketing data exist. However, in sub-Saharan Africa, the treatment-seeking pattern for maladies including malaria is complex and impacted by the local health system, culture and resources of the patient [6,7]. Anti-malarials are often purchased from private retail sector without a confirmed diagnosis of malaria. These vendors have been identified as an important source of medicines and health care close to people's homes [8] and thus complement formal health services. Information on the volume and types of medicine purchased from these vendors and specifically the level of exposure to anti-malarials is not available. Establishment of a simple practical system for surveillance from private drug outlets will be a useful tool for pharmacovigilance and tracking possible adverse drug reactions. With the recent introduction of ACT and distribution of millions of doses in sub-Saharan Africa, there is concern about adequately monitoring ADR in the population [9]. This study was designed to assess exposure to anti-malarial drugs in Sagamu community and to explore the use of widely available technology as a tool to monitor ADRs following exposure of malaria patients to antimalarial drugs.

## Methods

### Study setting

Sagamu, located 50 km north of Lagos is the seat of the Sagamu Local Government Area, Ogun State in south-west Nigeria. The town is spread over 614 Km^2 ^(237/Sqm) with an estimated population of 228,382. A large proportion of the population commutes to the city of Lagos daily for work or other commercial activities. Malaria is highly endemic in the area, accounting for most outpatient visits in the health facilities. Transmission occurs all year around with an upsurge in the rainy season - June to September [10]. The community is served by several schools, hospitals (Primary Health Care Centers, Private and Tertiary Hospitals), banks and hotels.

### Selection of study centers

Study centers were identified and selected from the list of registered pharmacies and Patent and Proprietary Medicine Stores (PPMS) compiled by the Department of Pharmaceutical Services, Ministry of Health, Ogun State. There are 128 PPMS and 13 pharmacies listed in the state's official records. The patient load in these facilities record average of 225 per day; (pharmacies, range 150-400 per day; PPMS, 35-50 per day).

From the 141 drug outlets a sample size of seven (three pharmacies and four PPMS) was determined at confidence interval of 50% and confidence level of 95% using Survey System sample size calculator http://www.surveysystem.com/sscalc.htm. The specific facilities were chosen using computer generated simple randomization procedure.

The study was descriptive and prospective, and involved active follow-up of sales of drugs in the selected centres for a period of four weeks (28 days) to determine the exposure to anti-malarial drug, measured by the amount of drugs purchased within the period. A drug-data chart was designed to capture information on category of drugs purchased. The proportion of anti-malarial of the different drugs purchased at the drug retail outlets, which at the time of study were not tracked by any active surveillance method, and sales per week of the medicines were determined. The anti-malarial drugs were classified to assess the drug use pattern for the different anti-malarial drugs and the rate of purchase between PPMS and the community pharmacies. The retail prices of the different anti-malarial drugs in a complete dosage pack were recorded.

The willingness of the buyers of the anti-malarial drugs in this community to participate in pharmacovigilance (PV), through a toll free mobile phone monitoring of adverse drug reactions, was evaluated in a total of 100 subjects who presented at the study sites to purchase anti-malarial drugs, volunteered to participate and provided information via mobile phones over a period of two weeks following purchase of medicine. The mobile phones were used to provide information on any ADRs experienced following ingestion of the anti-malarial drug, particularly ACT.

Prior to enrolment in the mobile phone ADR monitoring, the objective was explained to each of subjects after drug purchase was completed by a member of the study team and informed consent obtained.

The mobile phone number of the participant was obtained on permission and entered into a registration form. One member of the study team called the mobile number to validate correctness of entry and connectivity. A series of questions were prepared to inquire about any complaint or reactions observed by the subjects to whom the drug is administered, if adult, or a caregiver when subject is a child. Three of the investigators (SB, TA, AM) were involved in the documentation of any complaints or observations following drug use. Each participant is called from mobile phone designated for the exercise by a member of the study team. The participants mobile phones were called at specified times to record time of use of drug and 6-hourly for the first three days and 24 hourly from day 3 to day 14. All complaints or observations were recorded.

### Data collection

The pharmacies and medicine stores operate on an average of 13 hours daily (09.00 hr - 22.00 hr). Drugs purchased are recorded daily to monitor sales and to prevent stock-outs. The data on anti-malaria drug sales was collected daily from the outlets in the 28 days and transferred into a computer database. Information provided during mobile phone calls were transferred to forms designed for the purpose.

Following compilation of mobile phone interaction with patients or caregivers, each complaint or observation was examined and determined qualified for ADR. A complaint or response to inquiries by one of the research team member on phone is categorized as ADR from a patient if it qualifies to be 'any response to the drug which is noxious and unintended, and that occurs at doses normally used in man for prophylaxis, diagnosis, or therapy of diseases or modification of physiological function'; and an adverse event if 'any untoward medical occurrence present in a patient administered the medicine and which does not necessarily have to have causal relation with the treatment'.

### Data entry and analysis

Generic names, brand names (if possible), pharmacological classifications, collection centre, date of collection and day of study (0-27 days) were entered into a Database. The data generated were analysed using SPSS version 10 for Windows (SPSS Inc, Chicago USA). Chi-square analysis was used to compare proportions and linear regression analysis was used to obtain relationship between responses and duration of follow up for the monitoring aspect of this study.

### Ethics

Ethical approval for the study was obtained from the Joint Ethics Committee of the Olabisi Onabanjo University Teaching Hospital and Obafemi Awolowo College of Health Science, Olabisi Onabanjo University, Sagamu.

## Results

The study was carried out between May-September 2010. In the seven drug retail outlets, the distribution of drugs purchased in this community in the period of the study is shown in Table 1. Analgesics (15.8%). antibiotics (15.2%), vitamin supplements (13.3%), and anti-malarials (12.4%), respectively, had the highest frequencies of purchased drugs at these retail outlets. A total of 10,581 (87.5%) complete treatment dosages were sold at the pharmacies, while 1,512 (12.5%) complete treatment dosages were sold at the PPMS.

**Table 1:** Distribution of drugs purchased in the 4 weeks of drug surveillance in the retail outlets studied

Classes	Complete treatment dosages	Percentage (%)
Analgesics	1916	15.8

Antibiotics	1837	15.2

Anti-malarials	1500	12.4

Haematinics	870	7.2

Herbal preparations	71	0.6

NSAIDs	1273	10.5

Vitamins	1612	13.3

Others	(1-269)*	0.0-2.2

* = Purchase of other classes of drugs (e.g antidiabetics, haematinics and hormonal drugs) from these drug outlets ranges from 1 to 269 sales.

### Drug sales

Of 10,581 complete treatment dosage drugs purchased at the pharmacies, 10.7% were anti-malarials. Sales of anti-malarials at the PPMS were 23.7% of complete treatment dosages (1,521) sold. The weekly sales of anti-malarial and other categories of drugs from these outlets are shown in Table 2. On average, 375 complete treatment dosages of anti-malarial drugs were purchased weekly.

**Table 2:** Complete treatment dosages of anti-malarial drugs purchased per week during the active drug surveillance in the retail outlets

Weeks	Anti-malarials	Antibiotics	Analgesics	Vitamins	Total drug dosage consumed	Percentage (%)
1	434	443	476	455	3088	25.5
2	468	454	530	427	3208	26.6
3	365	556	432	431	3356	27.8
4	233	384	478	299	2441	20.2

### Types of anti-malarial and frequency of purchase

The various anti-malarial drugs that were available and frequently purchased from the study drug outlets were sulphadoxine-pyrimethamine (SP), chloroquine (CQ), artesunate (ART), artemether-lumefantrine (AL), amodiaquine (AQ), quinine (QN), herbal preparations (HP), halofantrine (HF), and proguanil (PR). Table 3 shows the distribution of anti-malarial drugs purchased as complete treatment dosage from the private retail drug outlets studied. The PPMS recorded higher purchases of SP (44.5%) and CQ (29.8%) compared to community pharmacies which had 37.7% and 23.7% for SP and CQ, respectively. However, artesunate (monotherapy) purchase was higher at community pharmacies compared with PPMS (18.1% vs. 9.7%, χ^2 ^= 13.6, P = 0.0002).

**Table 3:** The distribution of anti-malarial drugs purchased (in packs of a complete treatment dosage) from the private retail drug outlets studied

Anti-malarial drugs	Community Pharmacies (%) N = 1141	PPMS (%) N = 359
Sulphadoxine-pyrimethamine	430 (37.7)	160 (44.6)
Chloroquine	271(23.7)	107 (29.8)
Artesunate	207(18.1)	35 (9.7)
Artemether-Lumefantrine	117(10.3)	33 (9.2)
Amodiaquine	75 (6.6)	24 (6.7)
Quinine	28 (2.4)	0 (0)
Herbal preparations	7 (0.6)	0 (0)
Halofantrine	2 (0.1)	1 (0.2)
Proguanil	3 (0.2)	0 (0)

PPMS- Patent and Proprietary Medicine Stores

On average, artesunate was the most purchased anti-malarial at the pharmacies each week of the study period, but the least purchased at the PPMS. CQ was however the least purchased at the community pharmacies while it was the most purchased at the PPMS. The retail price per dose of anti-malarial drug (Table 4) varied with CQ being the cheapest and halofantrine, the most expensive.

**Table 4:** Cost of anti-malarial drugs at the time of the present study in the retail outlets

S/N	DRUGS	PRICES	
		NAIRA (N)	DOLLAR ($)
1	Sulphadoxine-pyrimethamine	140	0.9
2	Chloroquine	50	0.3
3	Artesunate	280	1.9
4	Artemether-Lumefantrine	700	4.7
5	Amodiaquine	150	1
6	Quinine	90	0.6
7	Herbal preparations	450	3
8	Halofantrine	1,150	7.7
9	Proguanil	500	3.3

### Mobile phone for monitoring of adverse drug reaction

**Figure 1:**
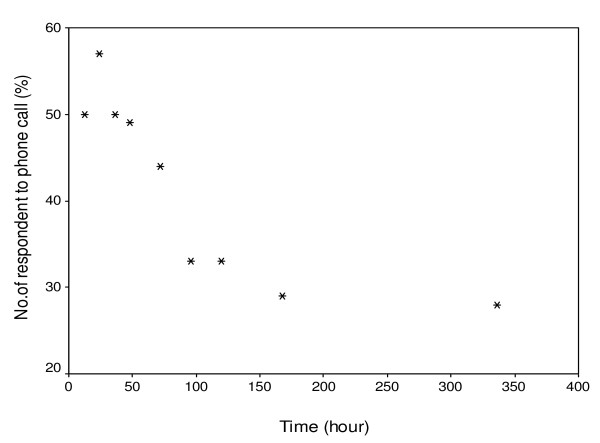
**Relationship between responses to toll-free mobile phone calls and time of call during monitoring of adverse drug reactions to anti-malarial drugs purchased in the retail outlets (r = 0.81, F = 13.7, P = 0.008)**.

100 volunteers (84 women and 26 men) participated in the toll free mobile phone monitoring of adverse drug reactions. 54% of the participants had between 6 and 12 years of formal education and 46% of the subjects had more than 12 years of formal education (post-secondary school), 5% of them were healthcare providers (nurses or doctors). The trend of the responses received showed that most people responded within the first twenty-four hours of follow-up. By 96 hours, the level of responses began to decline (Figure 1) with only few respondents completing the entire follow up period. No incidence of unknown ADRs was recorded and neither was there any increase in the frequency of known ADRs during the period of monitoring.

## Discussion

Exposure to anti-malarial drugs as a result of chemoprophylaxis or treatment of the infection and choice of drug depend on the clinical judgment of the physician, treatment policy, availability or cost. The adoption of ACT for malaria treatment and increasing access to this class of anti-malarial drugs offer an opportunity to evaluate occurrence of ADRs to these medicines in the larger population or community [5], Post-marketing surveillance offers assessment of drug released to the market in different categories of people, other than those in whom the drug was tested. ACT is 'new' to anti-malarial therapy in Nigeria and this is additional reason this study was carried to actively monitor possible ADRs over an extended period.

Data from this study indicate that medicines in general are purchased more from pharmacies compared to PPMS. However, a high volume of anti-malarial drugs (23.7%) is purchased at the PPMS. This implies that people in the study area, prefer PPMS as the first point of contact for anti-malarials than the pharmacies. Since the Alma Ata Declaration recognized the Primary Health Care (PHC) centres as positioned to be the first contact to individual, family and community, the frequency of visit of the people studied on PPMS for anti-malarials may suggest that the primary function of the PHC is not optimized in the study area. The centres are either underutilized or not easily accessible. The number of PPMS (128) and community pharmacies (13) in the community is a strong indicator of the challenges of PHC programme. PPMS appear more accessible given the high purchase rate for anti-malarial drugs recorded in this study and may be considered viable units for intervention programmes for malaria control in endemic areas, where PPMS exist. Negligence of these drug retail outlets may in turn, increase the risk of incorrect dosing, inappropriate treatment, impacting negatively on drug safety [11].

The large volume of analgesics may suggest the probability that most people at the point of purchasing anti-malarials also buy analgesics to relief the symptoms of malaria, which include fever and arthralgia. The observation during the study showed that over 50% of those purchasing anti-malarial drugs also bought analgesics.

The frequency of purchase of anti-malarials in communities will differ greatly and may influence serious ADRs, such as allergic and alveolitis. Despite continuing education by the Federal Ministry of Health, SP and CQ use still remains high despite widespread resistance reported in the area of study [12]; indicating that although ACT is the recommended first line treatment for malaria, the official malaria treatment policy is yet to be fully practiced in the study community, Similar studies conducted in other malaria endemic areas in Africa also show that continued use of ineffective anti-malarial drugs, especially due to poverty, remain a challenge [13,14]. It will, therefore, be necessary to improve strategies for the implementation of the policy on treatment of malaria and encourage the inclusion of community drug outlets level for policy compliance.

Resistance to CQ and failure of mono therapy in many endemic countries led to a widespread promotion of ACT [15]. Nevertheless, its adoption appears compromised within the study area with only 10% purchase rate for ACT. This may be due to the cost of the artemisinins being more expensive than the older anti-malarials, either as artesunate monotherapy or as a combination [15]. Thus the cost of ACT may compromise effective treatment of malaria in poor countries with high malaria intensity. The relatively high cost of ACT in spite of huge financial and logistic commitments to intervention programmes from donor agencies remains an enigma.

The monitoring of adverse reactions to these drugs should be an important component of the health care system. The challenges of pharmacovigilance in Africa are not unconnected to the poor reporting structures and lack of motivation to report. The present study evaluated the use of mobile phone technology for monitoring ADRs, especially knowing that the exposure to antimalarial is relatively high in the community. Finding from the study showed that mobile phones offer a practical means of reporting adverse reaction to anti-malarial drugs and may be a model of choice in Africa where mobile telephony penetration and coverage is already driving innovation in agriculture and commerce [16]. The enthusiasm to report ADRs in the volunteers, who participated in this study, was highest within 24 hours and dwindled after 96 h. Improved education or enlightenment may improve this pattern over time.

The aim of this research is to provide "proof of principle" of an innovative approach to support pharmacovigilance in Africa. With over 60% of Africans, in the cities and small villages owning mobile phones,[16] it is expected that response to monitoring ADRs in this part of the world can be improved.

It is noteworthy that retail sectors do serve as source of malaria treatment and care, complementary to health facility [17], however, a major concern in this study is the high exposure to ineffective anti-malarial drugs at these drug outlets. Education and monitoring of community drug outlets by the health authority for compliance may provide a pragmatic approach. Training on the use of anti-malarials and concept of its PV for these shop keepers may be an option for improving safety of anti-malarial drugs and the quality of services rendered at PPMS. Coupling of the training with appropriate rewards for good practice may improve their performances [18].

Although, there was neither increase in the frequency of known ADRs or new ADRs detected during the study, it is important to ensure continuing monitoring of anti-malarial drugs safety and scale up use of mobile phone technology to support pharmacovigilance of anti-malarial drugs.

## List of abbreviations

ADRs: Adverse drug reactions; AL: Artemether-lumefantrine; AQ: Amodiaquine; AS: Artesunate monotherapy; CP: Community Pharmacies; CQ: Chloroquine; HF: Halofantrine; NSAIDs: Non Steroidal Anti-inflammatory Drugs; PHC: Primary Health Care; PPMS: Patent and Proprietary Medicine Stores; PR: Proguanil; QN: Quinine; SP-Sulphadoxine-pyrimethamine; USD: United State Dollars

## Competing interests

The authors declare that they have no competing interests.

## Authors' contributions

AAA participated in the conception of the study, field collection of data, analysis and writing of the manuscript. BS, AT, MA, OO, MOA, and OAT participated in planning the study, field collection of data, entering of data and analysis, and writing of the manuscript. IAO- participated in the conception of the study, planning of field work and monitoring. OAO- participated in the conception of the study, planning of field work and monitoring. OOA - participated in the conception of the study, planning of field work and monitoring. TTA- participated in the conception of the study, technical analysis, evaluation and writing of script. FAF - participated in the conception of the study, technical analysis, evaluation and writing of script. OATO- participated in the conception of the study, technical analysis, evaluation and writing of script. All authors have read and approved the final manuscript.
